# Enhanced Muscle Activation Using Robotic Assistance Within the Electromechanical Delay: Implications for Rehabilitation?

**DOI:** 10.1109/TNSRE.2024.3419688

**Published:** 2024-07-10

**Authors:** Alex C. Dzewaltowski, Philippe Malcolm

**Affiliations:** Department of Biomechanics and the Center for Research in Human Movement Variability, University of Nebraska Omaha, Omaha, NE 68182 USA. He is now with the Scholl College of Podiatric Medicine, Rosalind Franklin University of Medicine and Science, North Chicago, IL 60064 USA; Philippe Malcolm is with the Department of Biomechanics and the Center for Research in Human Movement Variability, University of Nebraska Omaha, Omaha, NE 68182 USA

**Keywords:** Human-robot interaction, motor control, nervous system, neurological disease, response time, physical therapy, biomechatronics, exoskeletons

## Abstract

Robotic rehabilitation has been shown to match the effects of conventional physical therapy on motor function for patients with neurological diseases. Rehabilitation robots have the potential to reduce therapists’ workload in time-intensive training programs as well as perform actions that are not replicable by human therapists. We investigated the effects of one such modality that cannot be achieved by a human therapist: assistance and resistance within the electromechanical delay between muscle activation and muscle contraction during arm extension. We found increased muscle activation when providing robotic assistance within this electromechanical delay. Assistance provided within this delay moves the participant’s arm quicker than their own muscle and increases the subsequent peak voluntary muscle activation compared to normal arm extension by 68.97 ± 80.05% (*SE* = 0.021; *p* = 0.007). This is surprising since all previous literature shows that muscle activation either decreases or does not change when participants receive robotic assistance. As a consequence, traditional robotic rehabilitation incrementally reduces assistance as the patient improves to maintain levels of muscle activation which is suggested to be important for neuronal repair. The present result may enable therapists to no longer have to choose between providing assistance or increasing muscle activation. Instead, therapists may be able to provide assistance while also increasing muscle activation.

## I. Introduction

Reaching abilities are improved through exercise and repeated practice [1], [2], [3], [4]. Physical therapists implement these two central ideas, exercise and practice, when designing and implementing standard-of-care rehabilitation programs for patients with neurological diseases. Supervised exercise and repeated practice are time-intensive and, consequently, have inflated healthcare cost. In an effort to improve outcomes and reduce these costs, attempts have been made to leverage technological advances such as robotic devices, virtual reality, and at-home telehealth [5], [6], [7], [8], [9], [10]. Incorporation of these technology does not yet translate to improvements or decrements to movement outcomes compared to standard-of-care [5], [11], [12]. Instead, current evidence supports that these technologies expand the capacity for rehabilitation by facilitating some care from their homes or by reducing the number of hands-on hours required to provide therapy. These technological advances may be especially beneficial for patients that cannot complete the rehabilitation program without assistance from the physical therapist due to muscle weakness and impaired motor control [2], [13]. More specifically, robotic devices can potentially reduce the amount of time physical therapists need to dedicate to each patient, which may expand access to more patients [1], [2], [3], [4], [14].

With the inception of upper-arm robots such as the MIT MANUS in 1989, researchers looked to ‘optimize the delivery of therapy’ and ‘increase the productivity of caregivers’ [1], [2], [15], [16]. MIT MANUS is an upper-limb robotic rehabilitation device designed originally for patients following stroke. A key concept behind the MIT MANUS is that the assistance to the patient can be modified to provide the minimum amount of assistance necessary for the patient to perform repetitive movement practice. Once a patient can perform the task independently, the robotic rehabilitation device is programmed to only support the arm’s weight. This is done to maintain a consistent challenge during the repeated reaching tasks. Robotic rehabilitation that tailors down assistance can improve upper-limb function of patients, as measured by the Fugle-Meyer, upper limb score, compared to a group that did not receive robotic rehabilitation [16], [17], [18], [19], [20], [21], [22], [23], [24]. Using this concept for robotic devices, other groups with similar devices have also found similar positive results [18], [19], [20], [22], [23], [24]. This form of robotic rehabilitation can also improve physical therapy’s effectiveness for a wider variety of neurological deficits such as stroke, multiple sclerosis, cerebral palsy, or spinal cord injury [2], [18], [21], [23], [25]. However, robotic rehabilitation devices have yet to achieve rehabilitative gains that surpass conventional physical therapy [18], [26]. Specifically, the MIT MANUS robotic gym (which included wrist and hand modules) was compared to an enhanced upper limb therapy program and typical National Health Service care in the clinical trial RATULS consisting of 770 stroke patients divided between the groups [27]. Robotic rehabilitation and enhanced upper limb therapy did not improve upon usual care as measured by the Action Research Arm Test (ARAT); instead, all groups improved similarly [27].

Traditional robotic devices, like the MIT MANUS, provide assistance using an impedance controller with force feedback. This form of assistance relies on mechanical sensation to facilitate rehabilitation. While not the focus in those studies, mechanical sensation itself has been investigated for its efficacy as a neurological intervention because of the interaction between mechanical sensation and muscle activation. Increasing muscle activation - activation through the neuron - seems to be critical to the development and maintenance of axons [28], [29], [30], [31], [32], [33]. Layne et al. reported increases in muscle activation in response to pressure underneath the foot [34], [35], [36]. Localized vibration or small discrete mechanical stimulations have been applied to the sole of the foot to modify lower-limb muscle activation [34], [35], [36], [37], [38]. In the case of a discrete mechanical stimulus applied to the sole prior to the onset of voluntary muscle activation, there was an increase in subsequent muscle activation in the tibialis anterior and soleus [36]. The increase in muscle activation was attributed to the excitation of cutaneous afferents [35], [36], [37], [38]. However, the mechanical sensation has yet to be investigated using a robotic device that has more control of the timing and forces applied to the participant. Robotic devices are an accurate means to investigate the potential role of mechanical sensation and its effects on the nervous system. Specifically, the *timing* of force application (mechanical sensation) may play a role in a robotic device’s rehabilitative effectiveness.

The electromechanical delay between muscle activation and muscle contraction is ∼40ms [39], [40]. Delays that are significantly longer are indicators of neurological disease [41]. We have developed a robotic device framework to apply rapid pulls to a participant’s arm using a tethered actuator within the electromechanical delay between muscle activation and contraction. This rapid pull begins to move the participant’s arm in response to muscle activation sooner than the muscle itself begins to contract. By applying robotic, mechanical sensation within this delay, there could be a resulting superimposition of sensory afferent activation onto already engaged voluntary muscle activation. If the sensory feedback diverges from the goal of the engaged movement (a perturbation), it may increase the muscle activation required to complete the task. Again, increasing muscle activation - activation through the neuron - seems to be critical to the development and maintenance of the health of our nervous system and is beneficial even if the activation came from an externally applied electrical stimulus [28], [29], [42], [43], [44].

Functional electrical stimulation (FES) has been applied independently and in tandem with robotic rehabilitation to improve motor function in patients with neurological disease [42], [45], [46], [47]. This is partly due to inadequate recruitment of agonist muscles for movement [19], [48], [49], [50]. However, an increase in the activation along the axon paired with proprioception (mechanical sensation) seems to enhance a neuromotor intervention’s effectiveness greatly. Both the increase in activation and sensation trigger the maintenance, development, and selection of neuronal pathways within the nervous system [31], [32], [33], [51], [52], [53], [54], [55], [56], [57]. An impactful example can be found in research that applied electrical stimulation to the spinal cord of participants with permanent locomotor deficits [58]. In that study, participants’ body weight was supported during treadmill walking, and their spinal cord was electrically stimulated in appropriate regions to activate relevant lower limb muscles. The timing of the electrical stimulation coincided with the muscle group’s typical activation phase during typical, unaffected walking. By applying the electrical stimulus within the appropriate phase of activation, participants’ walking abilities improved in a significantly shorter time compared to a similar protocol that simply applied a continuous electrical stimulus [58], [59]. By applying electrical stimulation at appropriate timings, proprioceptive information transmission was not blocked from foot contacts in contrast to a continuous stimulation. The effectiveness of the electrical stimulation at the spinal cord greatly improved patients’ walking abilities when retaining the proprioceptive information in the form of force sensation at the foot. This highlights the potential importance of mechanical sensation, not only for increasing the activation of neurons, but also for improving the neuromotor function of patients [43]. A robotic device that increases muscle activation using a mechanical sensation may be a beneficial option for the rehabilitation of patients with neurological disease. The aim of the present study was to evaluate the effects of an assistive or resistive actuator pull with onset timings defined relative to muscle activation.

## II. Methods

Fourteen healthy, young participants were recruited (age: 25.43 ± 3.32yrs, height: 174.50 ± 9.89cm, weight: 73.51 ± 13.44kg, males = 8). The number of participants was considered similar to previous studies that implemented robotic rehabilitation [17]. The University of Nebraska Medical Center’s Institutional Review Board approved the study protocol. Participants completed a self-report of health history confirming that they were free of any neurological, musculoskeletal, or cardiovascular impairment that would limit their ability to complete repeated arm extensions.

**Fig. 1.**
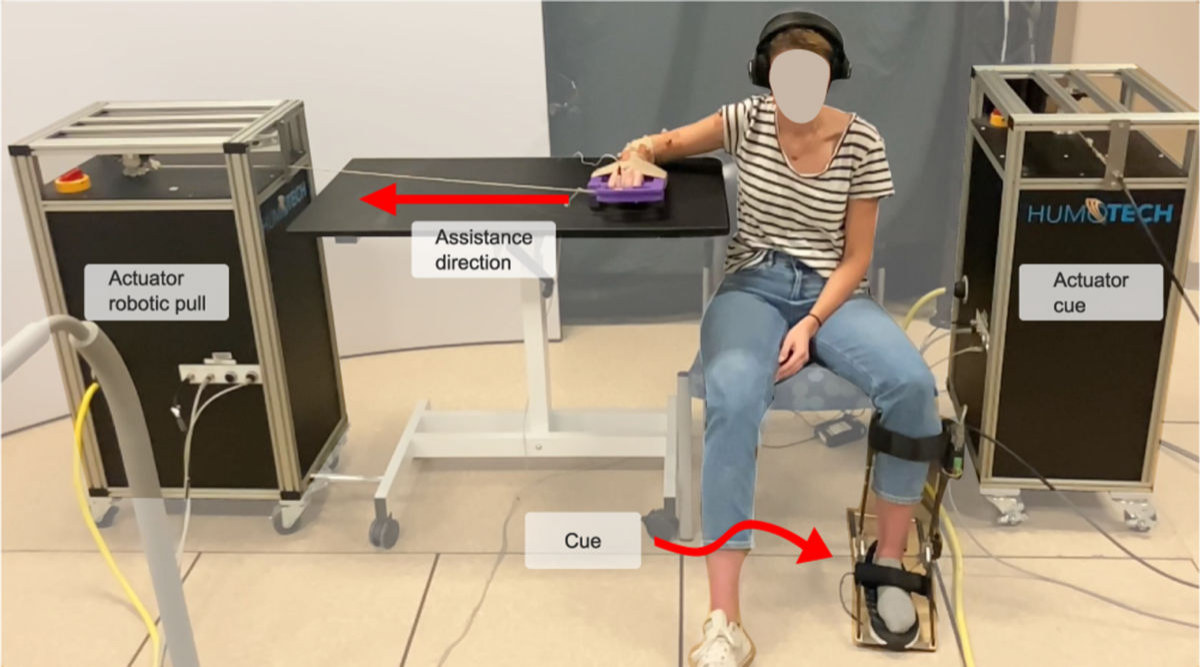
Example equipment set up for assistive actuator pull. The cue, or the push underneath the participant’s foot, is performed by the actuator on the right via a Bowden cable and HuMoTech ankle exoskeleton end effector. The left actuator provides assistive pulls via a rotation sequence triggered by voluntary muscle activation. The actuator is moved to the other side of the participant to provide perturbative pulls.

Participants performed a rapid reaching task using a custom setup that could either assist or resist reaching using a single actuator (Fig. 1). The actuator unit is from a commercial cable robot system (HuMoTech, PA). Each participant sat with their right arm resting on a table while wearing noise-canceling headphones. The table’s height was raised or lowered such that the participant’s shoulder was near 80° of abduction. Slight position adjustments were made based on verbal interaction with the participant to ensure their comfort. This position was not changed during the collection. Participants gripped a custom handle with wrist support tethered to an actuator and performed each extension with the custom handle touching the table.

**Fig. 2.**
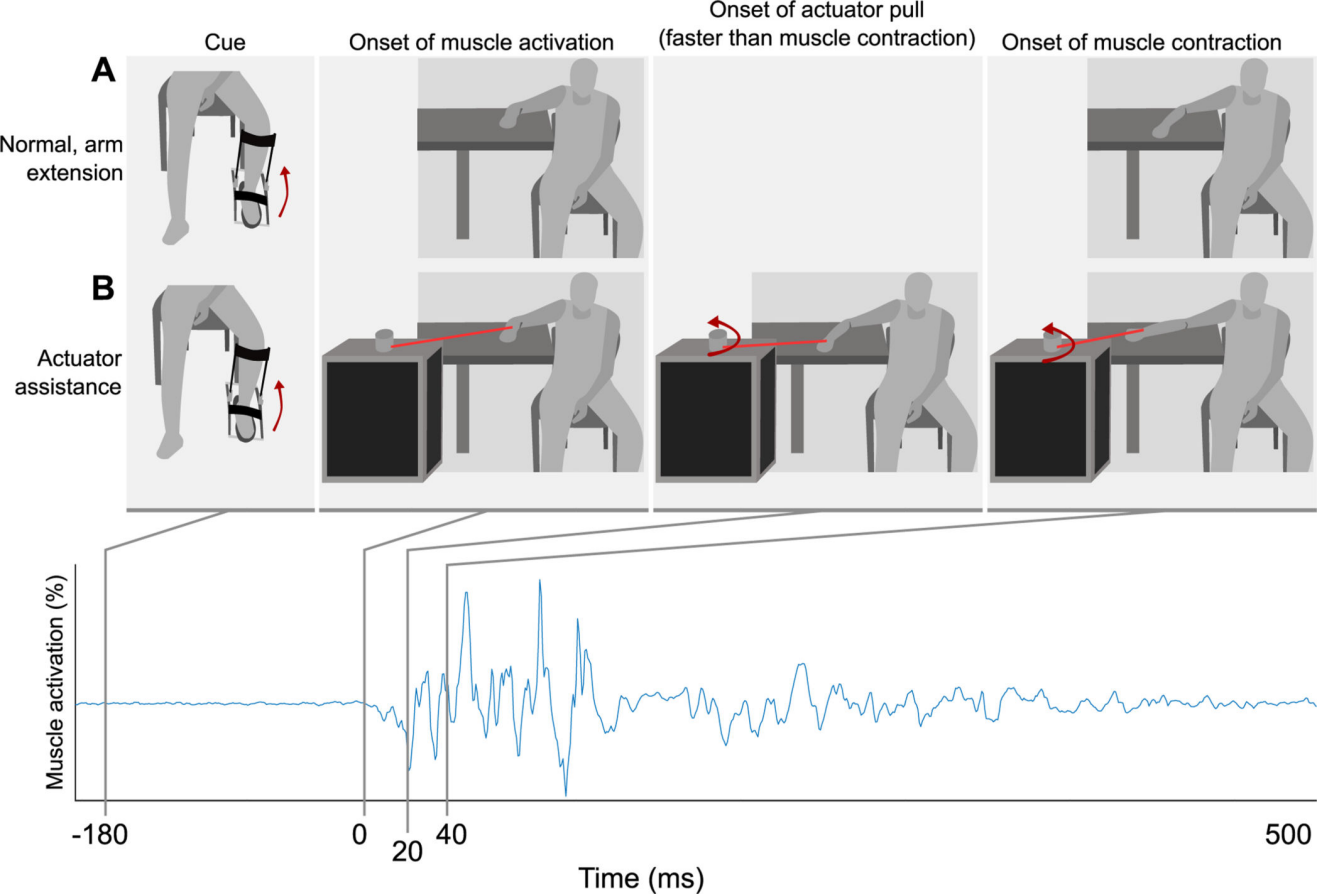
Explanation of rapid (faster-than-biological) assistance method. A) Control condition. Figure sequence showing how the cue is followed by the onset of muscle activation after a reaction time of about 180ms and onset of muscle contraction approximately 40ms after the activation onset due to the biological electromechanical delay. B) Rapid assistance condition. Our system setup detects muscle activation onset and begins to rotate the actuator approximately 20ms after activation onset resulting in a faster onset of arm movement than the control condition. After the normal biological electromechanical delay, the biological muscle also starts to contract while still being assisted by the actuator.

Our custom setup used surface electromyography (EMG, Noraxon, Scottsdale, AZ) placed on the agonist muscle for arm extension (e.g., triceps brachii) to trigger an actuator rotation sequence that was different for each condition. Dual-lead, wired surface electrodes were placed on the lateral portions of the triceps and biceps brachii. To trigger an actuator rotation sequence, a custom algorithm detected the onset of voluntary muscle activation from the triceps brachii that participants produced in response to feeling the push underneath their foot (Fig. 2). Our detection algorithm sets the onset timing to be 1% above the previous maximum squared EMG sampled from the previous rest period between individual arm extensions. The rest period between individual arm extensions was between 5–15s. While infrequent, this occasionally resulted in trials where the actuator pull was slower than intended due to participant movement being close to the timing of the cue, which raises the threshold used to detect onset.

Participants were instructed to extend their arm as quickly as possible as soon as they felt a small push underneath their left heel. The push underneath their foot, or the cue, was set to raise the heel by approximately 2cm. There was a cue for every arm extension, and the time between cues for each arm extension was randomized between 5–15s. Ten participants completed 250 arm extensions across five conditions, and four participants completed 300 arm extensions across six conditions, where these four participants also completed an additional passive condition.

The experimental conditions consisted of actuator pulls with two different timings and directions as well as two reference conditions: control and passive. The six different actuator conditions were named control condition, rapid assistance, delayed assistance, rapid perturbation, delayed perturbation, and passive assistance. The rapid conditions are the fastest our detection algorithm could achieve real-time triggering of the actuator following EMG onset with a delay of 23.4 ± 7.22ms (Fig. 2). The delayed conditions are a 40ms artificial delay added to our fastest detection for a total delay of 72.48 ± 15.43ms. The control condition consisted of arm extension without pulls from the actuator. Assistance conditions consisted of actuator pulls in the same direction as arm extension for 0.31s. Perturbation conditions consisted of actuator pulls in the opposite direction as arm extension for a duration of 0.1s. The passive assistance condition was performed by only four participants. For the passive condition, participants were instructed to relax and not perform arm extension once cued, though a pull was still provided in the assistive direction. Following the assistive pull, participants were instructed to return their arm to the starting position. The rotational speed for each actuator sequence was constant for every condition at 40rads^−1^ (rope retraction speed of approximately 0.79ms^−1^).

**Fig. 3.**
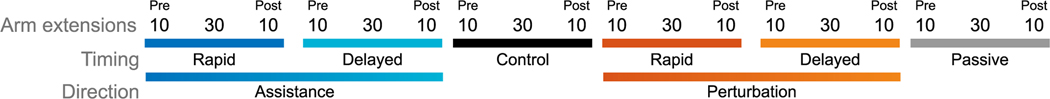
Example arm extension protocol. Conditions were performed in a randomized order except the passive condition was conducted last by four participants.

The order of conditions was randomized except for the passive condition, where the passive condition was always performed last. Each condition consisted of 10 arm extensions without a pull from the actuator, 30 arm extensions with a pull (except for the control condition), and 10 arm extensions without a pull, for a total of 50 arm extensions (Fig. 3).

In post-processing, biceps and triceps brachii EMG were rectified, high pass filtered with a cutoff frequency of 20Hz, and then low pass filtered with a cutoff frequency of 10Hz [60]. Participant data were normalized to the average peak EMG from the control condition. Motion capture of the arm was recorded with 16 cameras (Vicon, Oxford, UK, 200Hz). A total of seven retroreflective markers were placed on the shoulder, anterior and posterior lateral portions of the upper arm, the lateral epicondyle of the humerus (i.e., the elbow), anterior and posterior lateral portions of the arm, and one between the radial and ulnar process (i.e., the wrist). The angular velocity of the arm was calculated based on the change in position of the wrist marker with respect to the elbow marker in the transverse plane. This simplification is appropriate because the participants kept their elbow and custom handle against a flat table surface.

Following each actuator condition, participants were asked to rate their perceived arm extension speed for the first 10 trials compared to the middle 30 trials as well as the last 10 trials. Responses corresponded to a number between 1–10 where 1 indicated that the subsequent trials were much slower, a 5 indicated that the subsequent trials were the same speed, and a 10 indicated that the subsequent trials were much faster than the first 10 trials they completed for that condition. These two questions were asked following the completion of every condition except for the passive condition:

*Q1*: Did you feel that you were much faster, much slower, or the same during the middle 30 trials as the first 10 trials you just completed?

*Q2*: Did you feel that you were much faster, much slower, or the same during the last 10 trials compared to the first 10 trials you just completed?

### A. Statistical Analyses

Linear mixed models were used to test for the significance of peak muscle activation of the triceps and biceps brachii within the first 200ms following EMG onset detection, reaction time, and each questionnaire item. Two fixed effects were evaluated, including one for each condition (excluding the passive condition, which was only performed by four participants) and one for the order in which each condition was conducted. One random effect was used for within-subject variance. A Tukey post hoc was used to evaluate the main effect of condition with a Bonferroni-Holm adjusted *α*-level of 0.05.

## III. Results

### A. Muscle Activation

**Fig. 4.**
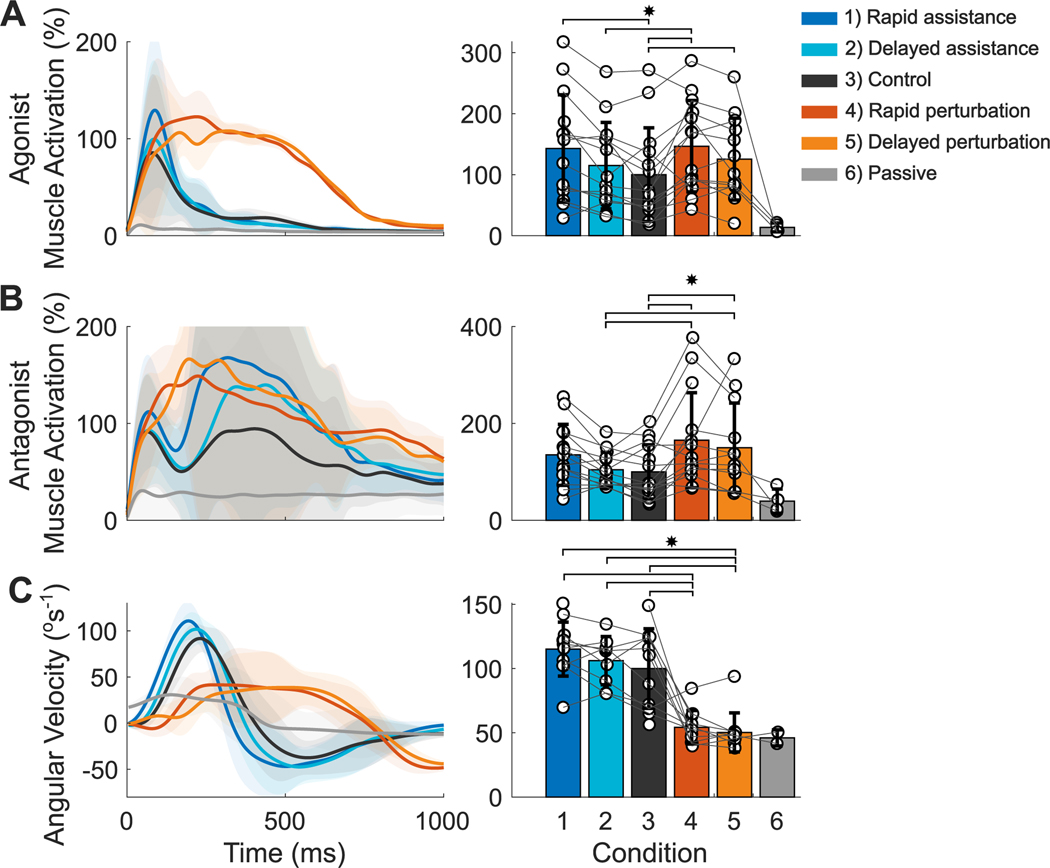
Muscle activation and kinematics. A) Agonist muscle activation (triceps brachii). B) Antagonist muscle activations (biceps brachii). C) Angular velocity of the wrist about the elbow. Negative values for angular velocity represent arm flexion. Lines indicate the mean muscle activation or angular velocity of each condition. The shaded regions indicate between-participant standard deviation. Bars represent the mean peak values. Error bars represent the between-participant standard deviation. White dots connected by lines are the same participant’s peak value during each condition. Muscle activation is reported as a percent normalized by mean peak muscle activation of the control condition. ^∗^Stars indicate significant differences (p < 0.05).

A significant main effect of condition was found in peak muscle activation of the triceps brachii (agonist) (*X*^2^(4, 14) = 21.15; *p* < 0.001, Fig. 4). Rapid assistance resulted in 68.97 ± 80.05% larger peak muscle activation than the control condition (*SE* = 0.021; *p* = 0.007). Rapid and delayed perturbations were 81.91 ± 115.37% and 92.11 ± 112.071% larger than the control condition (*SE* = 0.027, 0.027; *p* = 0.043, *p* = 0.001). Peak muscle activation during delayed perturbation was 45.98 ± 55.77% larger than delayed assistance (SE = 0.026, *p* = 0.045).

A significant main effect of condition was also found in peak muscle activation of the biceps brachii (antagonist) (*X*^2^ (4, 14) = 23.31; *p* < 0.001). Rapid and delayed perturbations were 100.35 ± 120.27% and 85.81 ± 91.71% larger than the control condition (*SE* = 0.006, 0.006; *p* = 0.018, *p* = 0.004). Rapid and delayed perturbation were also 67.47 ± 107.90 and 54.72 ± 57.03% larger than delayed assistance (*SE* = 0.006, 0.006; *p* = 0.002, *p* = 0.009).

### B. Reaction Time

Reaction time was defined as the duration between the cue and EMG onset. A main effect of condition was found on reaction time (*X*^2^ (4, 14) = 10.9, *p* < 0.03). Reaction time for delayed perturbation was 45.52 ± 65.04ms slower than rapid assistance (SE = 15.41, *p* < 0.031).

### C. Arm Velocity

A significant main effect of condition was found for peak arm angular velocity (*X*^2^ (4, 14) = 126.83; *p* < 0.001). Rapid and delayed perturbations were 39.51 ± 32.04% and 38.6 ± 28.85% slower than the control condition (*SE* = 31.185, 30.937; *p* < 0.001, *p* < 0.001). Rapid and delayed perturbations were 97.74 ± 54.29% and 94.38 ± 65.86% slower than delayed assistance (*SE* = 34.03, 32.67; *p* < 0.001, *p* < 0.001). Rapid and delayed perturbations were 129.15 ± 62.95% and 123.88 ± 70.85% slower than rapid assistance (*SE* = 30.37, 30.39; *p <*0.001, *p* < 0.001).

### D. Questionnaire

**Fig. 5.**
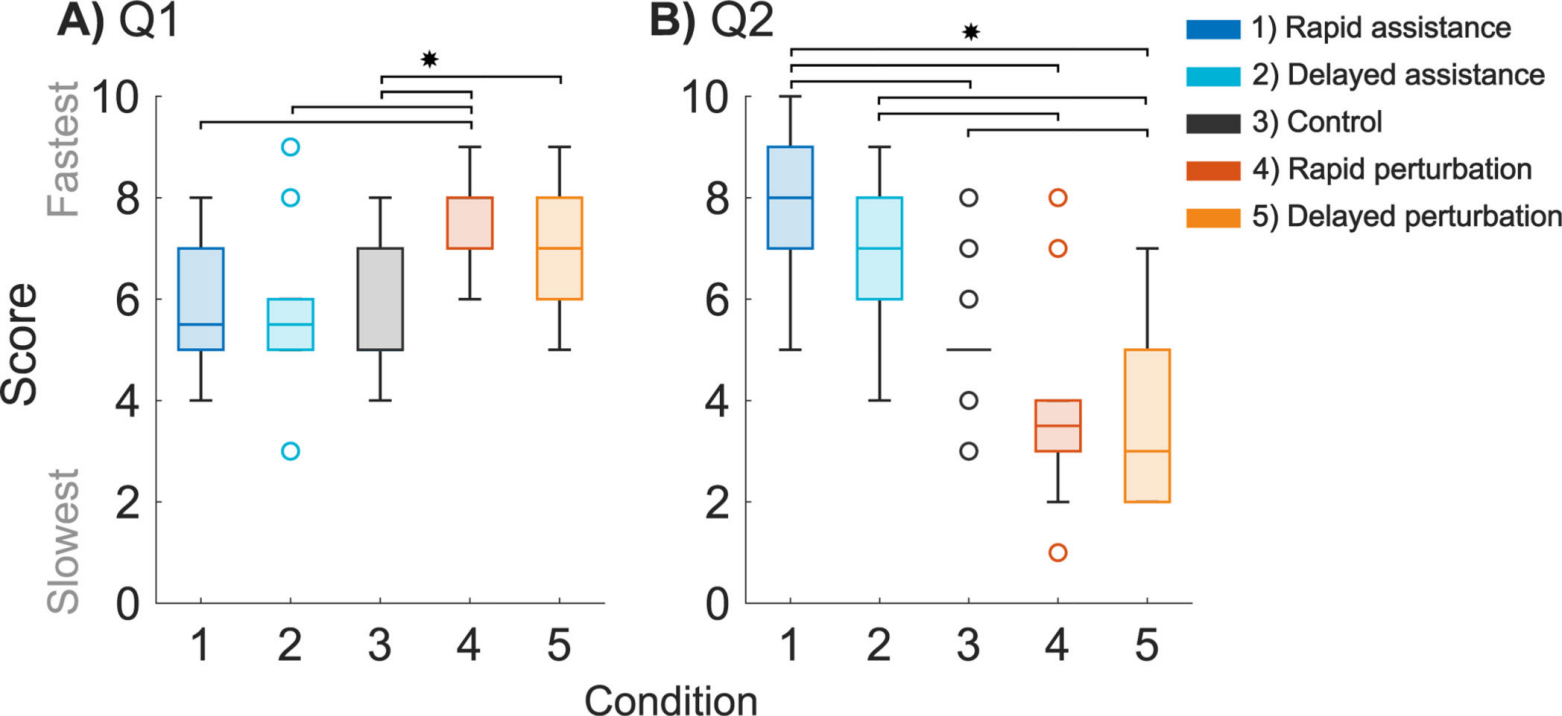
Questionnaire results A) Responses to questions that asked participants if they felt faster, slower, or the same following actuator pulls (Q1). B) Responses questions that asked participants if they felt faster, slower, or the same when receiving actuator pulls (Q2). ^∗^Stars indicate statistical significance (p < 0.05).

A significant main effect of condition was found for Q1 (*X*^2^ (4, 14) = 30.308; *p* < 0.001, Fig. 5). Rapid and delayed perturbation felt slower than the control condition when receiving pulls from the actuator relative to the pre-extension trials (*SE* = 0.434, 0.438; *p* = 0.033, *p* < 0.001). Rapid and delayed assistance felt faster than the delayed perturbation when receiving pulls from the actuator relative to the pre-extension trials (*SE* = 0.435, 0.437; *p* = 0.004, *p* = 0.006).

A significant main effect of condition was found for Q2 (*X*^2^ (4, 14) = 69.038; *p* < 0.001). Rapid assistance felt faster than the control (*SE* = 0.610; *p* = 0.002), as well as the rapid and delayed perturbation when comparing pre to post extension trials (*SE* = 0.611, 0.610; *p* < 0.001. *p* < 0.001). Rapid and delayed perturbations felt slower than delayed assistance when comparing pre to post extension trials (*SE* = 0.626, 0.608; *p* < 0.001. *p* <0.001). Rapid perturbation felt slower than the control condition (*SE* = 0.612; *p* = 0.033).

## IV. Discussion

Participants performed arm extension with resistance and assistance provided by a tethered actuator within the electromechanical delay between muscle activation and muscle contraction. We investigated the significance of applying mechanical resistance and assistance with this specific timing on muscle activation, kinematics, and reaction speed in reference to the control and passive conditions. Furthermore, we considered participants’ perceptions about the different actuator conditions. Rapid assistive actuator pulls significantly increased muscle activation of triceps brachii during arm extension compared to the control condition (*p* = 0.007). This is surprising because robotic assistance normally reduces the muscle activation required for movement [61], [62], [63].

The increased muscle activation during rapid assistance could have been attributed to co-contraction or faster angular velocities; however, both antagonist activation (biceps brachii) and peak angular velocity did not significantly increase compared to the control condition (*p* = 0.329, 0.246). Furthermore, analysis of the passive condition shows that passively pulling the arm did not produce substantial activation. Specifically, the effect of the rapid assistance is far greater than the summation of activation due to a mechanical pull (passive condition) and a voluntary arm movement (control condition). Our results suggest that the quicker onset timing of rapid assistance is likely a key feature to the observed increase in agonist activation because agonist activation during delayed assistance was not significantly different from the control condition. For this surprising finding, we discuss three potential explanations: superimposition of sensory information, reduction in Golgi tendon inhibitory signaling, and eliciting a startle reflex.

The increased muscle activation during rapid assistance may have resulted from a superimposition of sensory information onto the already engaged voluntary muscle contraction [35], [36], [37], [38]. This would be in agreement with previous studies that applied pressure to the sole of the foot prior to voluntary contraction and found subsequent increases in muscle activation of the soleus and tibialis anterior [35], [36], [37], [38]. Mechanical sensation itself can excite functionally related neurons and, situationally, increase muscle activation in response to the stimulus [64], [65], [66].

As an alternative explanation, the unexpectedly faster onset of extension for the participant may have reduced muscle stretch and muscle fascicle force in the agonist muscle, thereby reducing inhibitory signaling from Golgi tendon organs. We confirmed that rapid assistance results in a faster onset of arm extension (this is inherent to the study design, rapid pulls move the arm sooner than delayed pulls), and therefore, rapid assistance may suddenly reduce muscle fascicle force [67]. Golgi tendon organs produce inhibitory neural signaling in response to muscle force development. As muscle force increases, Golgi tendon organs serve as a protective mechanism that prevents excessive force production or excessive muscle tension. A rapid decrease in muscle force could reduce the inhibitory signaling and, therefore, lead to an increase in muscle activation or activation in the motor neuron. This is not to be confused with the Golgi tendon reflex pathway. The Golgi tendon reflex pathway applies inhibitory signaling to the agonist and excitatory signaling to the antagonist as a protective mechanism in response to excessive muscle force [68], [69]. However, we did not find a significant increase in antagonist activation, and therefore, Golgi tendon organs may play a role in our findings but most likely only via inhibitory signaling.

An additional alternative hypothesis is that rapid assistance elicits a startle reflex superimposed on voluntary contraction [70]. Previous studies suggest that alternate neural connections, separate from the voluntary activation pathway, can activate muscles and are accessible via a startling sensory response [71], [72], [73]. Execution of the startle reflex may be related to a preplanned motor response that is involuntarily released via a startling stimulus [74]. In this case, the instructed arm extension is the preplanned motor response. For rapid assistance, the increase in muscle activation may be due to involuntary activation of the agonist muscle through an alternate neural pathway that is activated near simultaneously with the voluntary neural pathway [73].

The perturbation condition increased activation of agonist and antagonist muscles, but the peak angular velocity of the arm remained similar to the passive condition. Considering these effects together, our perturbation conditions present results that are expected due to similarities with resistive exercise [75]. The rapid perturbation significantly increased muscle activation compared to delayed assistance but not rapid assistance. This may further support the potential benefits of rapid assistance as a rehabilitative intervention for neurological disease because the magnitudes of activation achieved here were similar to low-intensity resistive exercise (the perturbation condition). However, we consider the rapid perturbation being similar to resistive exercise as a technical limitation of our study.

We observed minimal changes to reaction time except between the conditions that were the most different, rapid assistance and delayed perturbation. This was to be expected as each actuator condition was only conducted for 30 trials. We chose 30 trials based on pilot tests that suggested that this number of trials would not interfere with subsequent actuator conditions. For our study design that evaluated several actuator conditions within the same session, it was imperative that we minimize the potential for adaptation effects from one condition to affect subsequent conditions.

Due to the novel interaction between motor and sensory responses presented by our framework, we asked participants basic questions to evaluate their perception of the differences in the experimental conditions. Participants considered arm extension without an actuator pull to feel faster following rapid and delayed perturbation conditions (*p* < 0.001, *p* = 0.033). The removal of restriction to arm extension resulted in a feeling of being faster. Participants considered rapid assistance to be significantly faster and delayed perturbation to be significantly slower than arm extension without an actuator pull (*p* < 0.001, *p* = 0.033). Participants identified which actuator conditions resulted in ‘faster’ and ‘slower’ arm extension as a function of direction but were not consistently able to identify differences in timing.

Future work will investigate this timing of rapid assistance in clinical populations. While increasing muscle activation could be useful in many populations, it remains to be tested how well this would translate to outcomes in clinical populations. For some patient subsets, rapid assistance is likely to be ineffective. Patients with resting tremor or patients unable to extend their arm will not be able to engage rapid assistance. The methods outlined here for applying rapid assistance are sensitive to the resting baseline muscle activation but also require some minimum amount of voluntary activation in response to a cue to trigger the actuator pull. As a consequence, direct application of rapid assistance during gait would be challenging because it is difficult to identify and/or separate onset of muscle activation. For patients that can engage, rapid assistance, there may be rehabilitative benefits. Fundamentally, neuronal reconstruction, repair, modification, etc. is activity dependent. Rapid assistance increases the electrical potential that was measured using surface EMG which may mean rapid assistance increases neuronal activity. However, the physiological mechanism that causes the observed increase in muscle activation is still unclear and therefore, future work will look to establish if rapid assistance is useful for rehabilitation of neurological patients. In addition, it remains to be tested whether the observed increased activation during arm extension in response to a heel cue would also exist during more functional movements (e.g. arm movements to restore balance in response to a tripping or slipping).

## V. Conclusion

Rapid assistance that increases voluntary muscle activation may support a paradigm shift in the implementation of robotic rehabilitation. Previous studies report a decrease in muscle activation when administering assistance [61], [62], [63]. Current implementation of robotic rehabilitation requires tuning the amount of assistance provided to patients to match their abilities. Increasing motor task difficulty is ultimately required to maintain elevated amounts of activation (necessary to excite neuronal pathways and signal repair [31], [32], [33], [45], [47], [51], [53]). With rapid assistance, the task difficulty may not need to be modified to increase muscle activation. Therefore, rapid assistance may address the previous tradeoff between a patient’s ability to complete a motor task and the difficulty of the motor task. Furthermore, recent reports have pointed to the importance of including mechanical sensation when designing rehabilitation protocols [43]. Rapid assistance achieves an increase in muscle activation via mechanical sensory stimulus, which seems to be critical to the speed of recovery for patients with neurological disease [43].

## Supplementary Material

supp2-3419688

supp1-3419688
